# Supplementary material for: Acceptance of communication technology, emotional support and subjective well-being for Chinese older adults living alone during COVID-19: A moderated mediation model
Source: PLoS One. 2023 Sep 21;18(9):e0291806. doi: 10.1371/journal.pone.0291806 (PMC10513265; doi:10.1371/journal.pone.0291806)
Supplement: S1 File — (PDF) [file pone.0291806.s001.pdf]

Supplementary Material 1 – Factor Analysis for Gerontology Acceptance Scale

Factor analysis was done using Principle Axis Factoring and Varimax rotation. The Kaiser-Meyer-Olkin measure (KMO) was 0.91 and all KMO values for individual items were above .5 (Yong & Pearce, 2013). The Barlett's test of sphericity χ^2 (91) = 3282.41, p < .001. Two factors – Communication Technology Acceptance and Communication Technology Usability – were extracted, and both explained a cumulative variance of 62.85%. The table below shows the factor loadings after rotation using a significant factor criterion of .4. Item 'You would find technology is easy to use' was removed as it was a complex variable and 'Your financial status does not limit your activities when using technology' was removed from the final analysis as it was not significant in our model.

Factor Structure of The Gerontology Acceptance Scale: EFA With Varimax Rotation (n=293)

		Communication Technology Acceptance	Communication Technology Usability
1.	Using technology is a good idea	.82	
2.	You like the idea of using technology	.79	
3.	Using technology would enhance your effectiveness in life	.88	
4.	Using technology would make your life more convenient	.86	
5.	You would find technology is useful in your life	.86	
6.	You would find technology is easy to use	.60	.53
7.	You could be skillful at using technology		.75
8.	You could complete a task using technology is there is someone to demonstrate how		.70
9.	You could complete a task using technology is you have just the instruction manual for assistance		.75
10.	You feel apprehensive about using the technology		.50
11.	You hesitate to use the technology for fear of making mistakes you cannot correct		.63
12.	You have the knowledge necessary to use the system		.79
13.	A specific person (or group) is available for assistance with technology difficulties		.59
14.	Your financial status does not limit your activities when using technology		
15.	When you want or need to use technologies, they are accessible for you		.65
16.	Your family and friends think/support that you should use technology		.65
	% of variance	10.91%	51.94%

Note: Factor loadings less than 0.32 have been omitted. Items 1-5 are for Communication Technology Acceptance, 7 - 16 are for Communication Technology Usability. EFA = exploratory factor analysis.

References

Yong, A. G., & Pearce, S. (2013). A beginner’s guide to factor analysis: Focusing on exploratory factor analysis. . *Tutorials in Quantitative Methods for Psychology*, *9*(2), 79-94.
