# Supplementary material for: Acceptance of communication technology, emotional support and subjective well-being for Chinese older adults living alone during COVID-19: A moderated mediation model
Source: PLoS One. 2023 Sep 21;18(9):e0291806. doi: 10.1371/journal.pone.0291806 (PMC10513265; doi:10.1371/journal.pone.0291806)
Supplement: Attachment — Submitted filename: The result of review PONE-D-22-27277.docx [file pone.0291806.s002.docx]

The result of review PONE-D-22-27277
Acceptance of Communication Technology, Emotional Support and Subjective Well-being for Chinese Older Adults Living Alone during COVID-19: A Moderated Mediation Model

This article is good, but there are some notes for improvement to make it better. First, describe the gap between the current condition and the desired condition. Second, describe the importance of using communication technology accompanied by supporting data. Third, add journals from similar previous studies. Passion for improvement. Goodluck.
