# Supplementary material for: Acceptance of communication technology, emotional support and subjective well-being for Chinese older adults living alone during COVID-19: A moderated mediation model
Source: PLoS One. 2023 Sep 21;18(9):e0291806. doi: 10.1371/journal.pone.0291806 (PMC10513265; doi:10.1371/journal.pone.0291806)
Supplement: Attachment — Submitted filename: Response to Reviewers.docx [file pone.0291806.s003.docx]

**Response to Editor Comments:**

**When submitting your revision, we need you to address these additional requirements.**

We thank the editor. We have addressed each comment below carefully and in detail. We believe the quality of the manuscript has improved and sincerely hope that PLOSOne will accept it for publication.

**Please ensure that your manuscript meets PLOS ONE's style requirements, including those for file naming. The PLOS ONE style templates can be found at** 
[**https://imsva91-ctp.trendmicro.com:443/wis/clicktime/v1/query?url=https%3a%2f%2fjournals.plos.org%2fplosone%2fs%2ffile%3fid%3dwjVg%2fPLOSOne%5fformatting%5fsample%5fmain%5fbody.pdf&umid=B1809329-F264-F405-A3DA-F12DF5FD7C14&auth=6e3fe59570831a389716849e93b5d483c90c3fe4-eb1b9d9024e7ff9d1ca49b2145a18dc936405229**](https://imsva91-ctp.trendmicro.com:443/wis/clicktime/v1/query?url=https%3a%2f%2fjournals.plos.org%2fplosone%2fs%2ffile%3fid%3dwjVg%2fPLOSOne%5fformatting%5fsample%5fmain%5fbody.pdf&umid=B1809329-F264-F405-A3DA-F12DF5FD7C14&auth=6e3fe59570831a389716849e93b5d483c90c3fe4-eb1b9d9024e7ff9d1ca49b2145a18dc936405229) **and** 
[**https://imsva91-ctp.trendmicro.com:443/wis/clicktime/v1/query?url=https%3a%2f%2fjournals.plos.org%2fplosone%2fs%2ffile%3fid%3dba62%2fPLOSOne%5fformatting%5fsample%5ftitle%5fauthors%5faffiliations.pdf&umid=B1809329-F264-F405-A3DA-F12DF5FD7C14&auth=6e3fe59570831a389716849e93b5d483c90c3fe4-c8caf757ed3bc401c4deea764d57e7f96d3050f0**](https://imsva91-ctp.trendmicro.com:443/wis/clicktime/v1/query?url=https%3a%2f%2fjournals.plos.org%2fplosone%2fs%2ffile%3fid%3dba62%2fPLOSOne%5fformatting%5fsample%5ftitle%5fauthors%5faffiliations.pdf&umid=B1809329-F264-F405-A3DA-F12DF5FD7C14&auth=6e3fe59570831a389716849e93b5d483c90c3fe4-c8caf757ed3bc401c4deea764d57e7f96d3050f0)

The manuscript has been rechecked to ensure the manuscript meets PLOS ONE’s style requirements.

**2. Please provide additional details regarding ethical approval in the body of your manuscript. In the Methods section, please ensure that you have specified the name of the IRB/ethics committee that approved your study.**

We have included the following paragraph stating the IRB/ethics committee that approved the study, as well as the approval number at the start of the methods section. (Page 6, line 133 to 134)

“The study has been approved by the National Healthcare Group (NHG) Domain Specific Review Board (DSRB), with DSRB approval number 2020/00945.”

**3. Please provide additional details regarding participant consent. In the Methods section, please ensure that you have specified (1) whether consent was informed and (2) what type you obtained (for instance, written or verbal). If your study included minors, state whether you obtained consent from parents or guardians. If the need for consent was waived by the ethics committee, please include this information.**

Written informed consent was obtained from all participants face-to-face prior to their participation in the study. More details have been included in the methods section. The following sentence has been included in the methods section. (Page 6, line 134 to line 136)

“In line with our ethics application, written informed consent was obtained from all participants face-to-face before data collection started.”

**4. We note that you have indicated that data from this study are available upon request. PLOS only allows data to be available upon request if there are legal or ethical restrictions on sharing data publicly. For more information on unacceptable data access restrictions, please see** [**https://imsva91-ctp.trendmicro.com:443/wis/clicktime/v1/query?url=http%3a%2f%2fjournals.plos.org%2fplosone%2fs%2fdata%2davailability%23loc%2dunacceptable%2ddata%2daccess%2drestrictions&umid=B1809329-F264-F405-A3DA-F12DF5FD7C14&auth=6e3fe59570831a389716849e93b5d483c90c3fe4-4cd0e449f6768c151c56e4f4df11540bced90dd4**](https://imsva91-ctp.trendmicro.com:443/wis/clicktime/v1/query?url=http%3a%2f%2fjournals.plos.org%2fplosone%2fs%2fdata%2davailability%23loc%2dunacceptable%2ddata%2daccess%2drestrictions&umid=B1809329-F264-F405-A3DA-F12DF5FD7C14&auth=6e3fe59570831a389716849e93b5d483c90c3fe4-4cd0e449f6768c151c56e4f4df11540bced90dd4)**.**

**In your revised cover letter, please address the following prompts:**

**a) If there are ethical or legal restrictions on sharing a de-identified data set, please explain them in detail (e.g., data contain potentially sensitive information, data are owned by a third-party organization, etc.) and who has imposed them (e.g., an ethics committee). Please also provide contact information for a data access committee, ethics committee, or other institutional body to which data requests may be sent.**

**b) If there are no restrictions, please upload the minimal anonymized data set necessary to replicate your study findings as either Supporting Information files or to a stable, public repository and provide us with the relevant URLs, DOIs, or accession numbers. For a list of acceptable repositories, please see** [**https://imsva91-ctp.trendmicro.com:443/wis/clicktime/v1/query?url=http%3a%2f%2fjournals.plos.org%2fplosone%2fs%2fdata%2davailability%23loc%2drecommended%2drepositories&umid=B1809329-F264-F405-A3DA-F12DF5FD7C14&auth=6e3fe59570831a389716849e93b5d483c90c3fe4-55f880e6bf0829d6a3ccf08331d6f4fa526c5561**](https://imsva91-ctp.trendmicro.com:443/wis/clicktime/v1/query?url=http%3a%2f%2fjournals.plos.org%2fplosone%2fs%2fdata%2davailability%23loc%2drecommended%2drepositories&umid=B1809329-F264-F405-A3DA-F12DF5FD7C14&auth=6e3fe59570831a389716849e93b5d483c90c3fe4-55f880e6bf0829d6a3ccf08331d6f4fa526c5561)**.**

**We will update your Data Availability statement on your behalf to reflect the information you provide.**

We have responded to the data availability statement in the cover letter via the following paragraph.

*“With regards to our data availability, we thank you for raising this issue. Unfortunately, access restrictions apply to the data underlying the findings due to national data protection laws and restrictions imposed by the ethics board to ensure data privacy of study participants. However, anonymous minimal dataset underlying the findings in the manuscript are available upon request after authorisation by the National Health Group Domain Specific Review Board. Interested researchers may contact the Geriatric Education and Research Institute Department of Policy and Governance at* [*enquiry@geri.com.sg*](mailto:enquiry@geri.com.sg)*.”*

**5. Please include your full ethics statement in the ‘Methods’ section of your manuscript file. In your statement, please include the full name of the IRB or ethics committee who approved or waived your study, as well as whether or not you obtained informed written or verbal consent. If consent was waived for your study, please include this information in your statement as well.**

The full ethics statement has been included in the methods section (Page 6, line 133 to 136). The statement includes the full name of the IRB or ethics committee who had approved the study. Written informed consent was obtained from all participants prior to the start of data collection and has also been included in the statement. Please refer to questions 2 and 3 for more details.

**6. Please include captions for your Supporting Information files at the end of your manuscript, and update any in-text citations to match accordingly. Please see our Supporting Information guidelines for more information:** [**https://imsva91-ctp.trendmicro.com:443/wis/clicktime/v1/query?url=http%3a%2f%2fjournals.plos.org%2fplosone%2fs%2fsupporting%2dinformation&umid=B1809329-F264-F405-A3DA-F12DF5FD7C14&auth=6e3fe59570831a389716849e93b5d483c90c3fe4-bfbea7a50952def9fde8cbe82f320c0d515ded70**](https://imsva91-ctp.trendmicro.com:443/wis/clicktime/v1/query?url=http%3a%2f%2fjournals.plos.org%2fplosone%2fs%2fsupporting%2dinformation&umid=B1809329-F264-F405-A3DA-F12DF5FD7C14&auth=6e3fe59570831a389716849e93b5d483c90c3fe4-bfbea7a50952def9fde8cbe82f320c0d515ded70)**.**

Captions in the supporting information files at the end of the manuscript have been included, and the in-text citations have been matched accordingly. For this manuscript, there is only one supporting file – S1_Factor Analysis for GAS.pdf

The citation for the supporting file has been included in the text (Page 8, line 180)

“…The EFA results for the analysis are available in S1 Appendix.”

The following section on Supporting information has also been included at Page 26, line 554.

**“S1 Appendix. Factor Analysis for GAS.”**

**7. Please review your reference list to ensure that it is complete and correct. If you have cited papers that have been retracted, please include the rationale for doing so in the manuscript text, or remove these references and replace them with relevant current references. Any changes to the reference list should be mentioned in the rebuttal letter that accompanies your revised manuscript. If you need to cite a retracted article, indicate the article’s retracted status in the References list and also include a citation and full reference for the retraction notice.**

The full reference list has been checked and updated accordingly. Changes are as follows:

Addition of the following papers in response to reviewers’ comments regarding (1) the importance of maintaining social communication for emotional support and SWB, (2) the effectiveness of using CT for SWB and (3) implications of our findings and competency on emotional support and SWB for older adults.

*5.	Zaninotto P, Iob E, Demakakos P, Steptoe A. Immediate and Longer-Term Changes in the Mental Health and Well-being of Older Adults in England During the COVID-19 Pandemic. JAMA Psychiatry. 2022;79(2):151-9. doi: 10.1001/jamapsychiatry.2021.3749. (Page 3, Line 63)*

*6.	Noguchi T, Hayashi T, Kubo Y, Tomiyama N, Ochi A, Hayashi H. Living Alone and Depressive Symptoms among Older Adults in the COVID-19 Pandemic: Role of Non–Face-to-Face Social Interactions. Journal of the American Medical Directors Association. 2023;24(1):17-21.e4. doi:* [*https://doi.org/10.1016/j.jamda.2022.10.014*](https://doi.org/10.1016/j.jamda.2022.10.014)*. (Page 3, Line 63)*

*7.	Savage RD, Wu W, Li J, Lawson A, Bronskill SE, Chamberlain SA, et al. Loneliness among older adults in the community during COVID-19: a cross-sectional survey in Canada. BMJ Open. 2021;11(4):e044517. doi: 10.1136/bmjopen-2020-044517.*

[*https://doi.org/10.1186/s41256-020-00154-3*](https://doi.org/10.1186/s41256-020-00154-3) *(Page 3, Line 63)*

*14.	Li J, Zhou X. Internet use and Chinese older adults’ subjective well-being (SWB): The role of parent-child contact and relationship. Computers in Human Behavior. 2021;119:106725. doi:* [*https://doi.org/10.1016/j.chb.2021.106725*](https://doi.org/10.1016/j.chb.2021.106725)*. (Page 4, Line 82)*

*15.	Kotwal AA, Holt-Lunstad J, Newmark RL, Cenzer I, Smith AK, Covinsky KE, et al. Social Isolation and Loneliness Among San Francisco Bay Area Older Adults During the COVID-19 Shelter-in-Place Orders. J Am Geriatr Soc. 2021;69(1):20-9. Epub 20201009. doi: 10.1111/jgs.16865. PubMed PMID: 32965024; PubMed Central PMCID: PMCPMC7536935. (Page 4, Line 85)*

*19.	Haase KR, Cosco T, Kervin L, Riadi I, O'Connell ME. Older Adults’ Experiences With Using Technology for Socialization During the COVID-19 Pandemic: Cross-sectional Survey Study. JMIR Aging. 2021;4(2):e28010. Epub 23.4.2021. doi: 10.2196/28010. PubMed PMID: 33739929. (Page 5, Line 108)*

*39.	Rodríguez MD, Gonzalez VM, Favela J, Santana PC. Home-based communication system for older adults and their remote family. Computers in Human Behavior. 2009;25(3):609-18. doi:* [*https://doi.org/10.1016/j.chb.2008.08.017*](https://doi.org/10.1016/j.chb.2008.08.017)*. (Page 16, Line 323)*

*40.	Yu RP, McCammon RJ, Ellison NB, Langa KM. The relationships that matter: social network site use and social wellbeing among older adults in the United States of America. Ageing and Society. 2015;36(9):1826-52. doi: 10.1017/s0144686x15000677. (Page 16, Line 323)*

*41.	Wu H-Y, Chiou A-F. Social media usage, social support, intergenerational relationships, and depressive symptoms among older adults. Geriatric Nursing. 2020;41(5):615-21. doi:* [*https://doi.org/10.1016/j.gerinurse.2020.03.016*](https://doi.org/10.1016/j.gerinurse.2020.03.016)*. (Page 16, Line 323)*

*44.	Hu Y, Qian Y. COVID-19, Inter-household Contact and Mental Well-Being Among Older Adults in the US and the UK. Frontiers in Sociology. 2021;6. doi: 10.3389/fsoc.2021.714626. (Page 16, Line 331)*

*45.	Choi NG, DiNitto DM, Marti CN, Choi BY. Telehealth Use Among Older Adults During COVID-19: Associations With Sociodemographic and Health Characteristics, Technology Device Ownership, and Technology Learning. Journal of Applied Gerontology. 2022;41(3):600-9. doi: 10.1177/07334648211047347. PubMed PMID: 34608821. (Page 16, Line 341)*

*46.	Vroman KG, Arthanat S, Lysack C. “Who over 65 is online?” Older adults’ dispositions toward information communication technology. Computers in Human Behavior. 2015;43:156-66. doi:* [*https://doi.org/10.1016/j.chb.2014.10.018*](https://doi.org/10.1016/j.chb.2014.10.018)*. (Page 16, Line 342)*

The following citations were removed as they were no longer applicable in the current version of manuscript:

*Mendes da Costa E, Pepersack T, Godin I, Bantuelle M, Petit B, Levêque A. Fear of falling and associated activity restriction in older people. results of a cross-sectional study conducted in a Belgian town. Arch Public Health 2012;70(1).*

*Taylor HO, Taylor RJ, Nguyen AW, Chatters L. Social Isolation, Depression, and Psychological Distress Among Older Adults. Journal of Aging and Health. 2018;30(2):229–46. doi:* [*https://doi.org/10.1177/0898264316673511*](https://doi.org/10.1177/0898264316673511)*.*

**8. In this article, there are some improvements that could be made to make it even better. The first step is to describe the gap between the current condition and the desired condition. Provide examples of how communication technology is used in conjunction with supporting data.**

We thank the editor for the comments. We have addressed the comments below.

a) We have more clearly identified the research gap and have inserted the following portion in the manuscript in page 6, line 126 to 130.

*“This study aims to address two main research gaps. Firstly, while older individuals who live alone have fewer chances for social interaction (e.g. Fingerman et al., 2021), few studies have examined how CT could impact SWB across different living arrangements. Next, both acceptance and competency in using CT are highly predictive of CT use [18] and provides additional information on how to address resistance and inertia in this population”*

b) With regards to providing examples on how communication technology is used in conjunction with supporting data. We have specified the type of communication technology used in this study, and highlighted its effectiveness on improving SWB. Please refer to the insertion below from the introduction section in page 4, Line 76 to 87.

*“… CT use via text-messaging has also been found to be as effective in providing emotional support as in-person interaction with close relational partners [13] during periods of stress.*

*During the early stages of the pandemic, CT use also proved effective in maintaining social communication when stringent safe distancing measures were in place. Reports from China suggest that using CT (e.g. via the internet) empowered older adults to maintain intergenerational contact, which also had a direct association with their overall SWB [14]. In a mixed-methods survey in San Francisco during shelter-in-place orders, participants who experienced less loneliness reported successful use of CT (e.g. telephone, video calls and internet) for social support [15]. It seems that the use of CT (e.g. phone calls and email) for social communication also buffered the risk of higher depressive symptoms, especially amongst older adults living alone [6].”*

**Response to Reviewers’ Comments**

**Reviewer #1: I'm wondering the study is relevant to current situation as it was conducted in pandemic situation in 2020. You need to validate your argument whether the findings can be useful theoretically and practically. I also would like to suggest the method section can be more detail, specifically for the study design and how the data was analysed.**

Thank you for your comments.

1a) We have restructured the discussion section to address your concerns. Theoretically, we believe that our study contributes to the existing literature by highlighting the moderating effect of living arrangements on the relation between CT use and SWB. Practically, CT use will become increasingly important with its proliferation in different aspects of life, including our daily activities and work beyond the pandemic in 2020. There is also a need to consider living arrangement when we think about which group of older adults to prioritise and target for CT education.

We have expanded on these points in the manuscript by including the following paragraphs from Page 16, line 321 to 342.

*“Our findings highlight the moderating effect of living arrangements on the relation between CT use and SWB. The association between CT, emotional support and SWB is prevalent in the literature [39-41]. However, these studies usually focus on the population, and few have assessed the effectiveness of those living alone vs living with others. Older adults living alone face more obstacles for social interaction, even during non-pandemic times. They report smaller social networks, receive less instrumental and emotional support [42] and lack of companionship [43]. Hence, it is important for older adults living alone to tap on CT as a tool to effectively engage in social communication [6, 14], especially during times of stringent social distancing. Meanwhile, for older adults living with others, our findings suggests that their SWB is not dependent on CT acceptance as they have options for face-to-face contact [4, 44] with other members of the household. However, CT use can still be encouraged to maintain contact and build relationships with others beyond the household.*

*Practically, there is a need to consider living arrangements when prioritising the different groups of older adults to target for CT education. Older adults living alone should be a priority as there is real potential for impact on their well-being during a pandemic situation. Beyond the pandemic, the prevalence of older adults living alone will increase, and as their physical function deteriorates, they will become less community ambulant. Thus, we will need to think about how to support their emotional health and the use of CT can be a key strategy. Programmes aimed at encouraging CT use amongst older adults should explicitly consider the role of CT acceptance and competency and how these might be enhanced by enablers such as encouragement from friends and families [21, 45], and access to CT devices and data plans [19], and reducing anxiety towards CT [46]. ”*

1b) We have also expanded the methods section to provide more details about our study design and data analysis approach. The following portions have been incorporated into the methods section.

Study Design: (Page 7, line 137 to 156)

*“The study used a cross-sectional study design. Data collection occurred between September to November 2020 via interview-administered surveys…Interested study participants were referred by senior activity centre (SAC) managers to be screened and recruited by the study team…Prior to the start of the survey, written informed consent was taken from all participants. Following which, participants responded to a 10-minute interview to aid recollection of their emotions and experiences during the circuit breaker (CB). Participants were encouraged to describe as vividly and as accurately as possible to aid recollection about that period. Following the interview was a 30-minute interviewer administered survey, where trained surveyors proceeded to collect participants’ background characteristics, CT acceptance and competency, emotional support and SWB measures. More details of the measures can be found in the following section.*”

Data Analysis: (Page 9, line 205 to 232)

*“Descriptive measures (i.e. frequencies, percentages) were populated for sociodemographic variables (e.g. age, gender, education) for all participants, participants living alone and participants living with others. Descriptive statistics (i.e. Mean, SD) for each variable was populated for all participants, participants living alone and participants living with others. Pearson correlations between the variables were also conducted to assess unadjusted associations between variables.*

*The first-step conditional mediation analyses were conducted via a two-step process using both Model 1 and Model 7 of the PROCESS macro [31]. For all models, age, education level and living accommodation were included as covariates due to their potential influence on CT acceptance and competency [32]. Language use was also included as a covariate as participants mentioned it as a contributing factor during the interviews. All predictor variables were centred by the PROCESS macro automatically to reduce multicollinearity.*

*Model 1 estimated the significance of the conditional effects between CT acceptance (competency), and emotional support based on living arrangements. With emotional support as the outcome (Y), we tested the effects of CT acceptance and CT competence (X) independently in two different models, which also included ‘living arrangement’ (0 = living with others, 1 = living alone) as moderator (W). To assess the significance of the moderating effects, we used 95% confidence intervals (CIs) based on 5,000 bootstrapped samples [33]. Simple slopes for the conditional effects were generated. Simple slopes were considered statistically significant if the corresponding 95% confidence interval did not include zero.*

*Following which, Model 7 was used to estimate the conditional indirect effects based on living arrangement. We ran a total of 6 models with either CT acceptance or CT competency as independent variables (X), emotional support as mediator (M), ‘living arrangement’ as moderator (W) and each of the three SWB outcomes (i.e. positive emotion, negative emotion, life satisfaction) as the dependent variable (Y). Significance of the model conditional indirect effects was determined by inferencing the 95% CIs based on 5,000 bootstrapped samples [33]. Indirect effects were considered statistically significant if the corresponding 95% confidence interval did not include zero.”*

**Reviewer #2: This article is good, but there are some notes for improvement to make it better. First, describe the gap between the current condition and the desired condition. Second, describe the importance of using communication technology accompanied by supporting data. Third, add journals from similar previous studies. Passion for improvement. Goodluck.**

Thank you for your comments.

2a) We have more clearly identified the research gap and have inserted the following portion in the manuscript in page 6, line 126 to 130

*“This study aims to address two main research gaps. Firstly, while older individuals who live alone have fewer chances for social interaction (e.g. Fingerman et al., 2021), few studies have examined how CT could impact SWB across different living arrangements. Next, both acceptance and competency in using CT are highly predictive of CT use [18] and provides additional information on how to address resistance and inertia in this population.”*

2b) Additional findings from the literature to address the importance of using CT supported by findings in the literature has been incorporated in two sections of the manuscript. The first section covers the impact of living arrangement on SWB, and how CT can be used as a tool to maintaining emotional support and SWB. This portion is included in the literature review. (Page 3, line 71 to line 87)

*“Past findings suggest that CT use can alleviate older adults’ social isolation by enabling connection with the outside world, gaining emotional support, and boosting self-confidence [10]. In addition, higher CT use was also associated with better self-rated health, higher SWB and fewer depressive symptoms through reduced levels of loneliness [11, 12]. Finally, CT use via text-messaging has also been found to be as effective in providing emotional support as in-person interaction with close relational partners [13] during periods of stress.*

*During the early stages of the pandemic, CT use also proved effective in maintaining social communication when stringent safe distancing measures were in place. Reports from China suggest that using CT (e.g. via the internet) empowered older adults to maintain intergenerational contact, which also had a direct association with their overall SWB [14]. In a mixed-methods survey in San Francisco during shelter-in-place orders, participants who experienced less loneliness reported successful use of CT (e.g. telephone, video calls and internet) for social support [15]. It seems that the use of CT (e.g. phone calls and email) for social communication also buffered the risk of higher depressive symptoms, especially amongst older adults living alone [6].”*

The second section is covered in the discussion section, where we further emphasise the use of CT not just as a tool for social communication, but its role in our daily lives in various settings such as work. (Page 16, line 321 to 342). Please refer to the response in section 1a) for more details.

2c) In addition, we have included additional literature from similar studies. As there are few studies that have studied this topic, we have included literature into various portions of the manuscript to cover the key topics including (1) the importance of maintaining social communication for emotional support and SWB, (2) the effectiveness of using CT for SWB and (3) implications of our findings of CT acceptance and competency on emotional support and SWB for older adults. The list of the papers included in order of appearance are as shown below.

*5.	Zaninotto P, Iob E, Demakakos P, Steptoe A. Immediate and Longer-Term Changes in the Mental Health and Well-being of Older Adults in England During the COVID-19 Pandemic. JAMA Psychiatry. 2022;79(2):151-9. doi: 10.1001/jamapsychiatry.2021.3749. (Page 3, Line 63)*

*6.	Noguchi T, Hayashi T, Kubo Y, Tomiyama N, Ochi A, Hayashi H. Living Alone and Depressive Symptoms among Older Adults in the COVID-19 Pandemic: Role of Non–Face-to-Face Social Interactions. Journal of the American Medical Directors Association. 2023;24(1):17-21.e4. doi:* [*https://doi.org/10.1016/j.jamda.2022.10.014*](https://doi.org/10.1016/j.jamda.2022.10.014)*. (Page 3, Line 63)*

*7.	Savage RD, Wu W, Li J, Lawson A, Bronskill SE, Chamberlain SA, et al. Loneliness among older adults in the community during COVID-19: a cross-sectional survey in Canada. BMJ Open. 2021;11(4):e044517. doi: 10.1136/bmjopen-2020-044517.*

[*https://doi.org/10.1186/s41256-020-00154-3*](https://doi.org/10.1186/s41256-020-00154-3) *(Page 3, Line 63)*

*14.	Li J, Zhou X. Internet use and Chinese older adults’ subjective well-being (SWB): The role of parent-child contact and relationship. Computers in Human Behavior. 2021;119:106725. doi:* [*https://doi.org/10.1016/j.chb.2021.106725*](https://doi.org/10.1016/j.chb.2021.106725)*. (Page 4, Line 82)*

*15.	Kotwal AA, Holt-Lunstad J, Newmark RL, Cenzer I, Smith AK, Covinsky KE, et al. Social Isolation and Loneliness Among San Francisco Bay Area Older Adults During the COVID-19 Shelter-in-Place Orders. J Am Geriatr Soc. 2021;69(1):20-9. Epub 20201009. doi: 10.1111/jgs.16865. PubMed PMID: 32965024; PubMed Central PMCID: PMCPMC7536935. (Page 4, Line 85)*

*19.	Haase KR, Cosco T, Kervin L, Riadi I, O'Connell ME. Older Adults’ Experiences With Using Technology for Socialization During the COVID-19 Pandemic: Cross-sectional Survey Study. JMIR Aging. 2021;4(2):e28010. Epub 23.4.2021. doi: 10.2196/28010. PubMed PMID: 33739929. (Page 5, Line 108)*

*39.	Rodríguez MD, Gonzalez VM, Favela J, Santana PC. Home-based communication system for older adults and their remote family. Computers in Human Behavior. 2009;25(3):609-18. doi:* [*https://doi.org/10.1016/j.chb.2008.08.017*](https://doi.org/10.1016/j.chb.2008.08.017)*. (Page 16, Line 323)*

*40.	Yu RP, McCammon RJ, Ellison NB, Langa KM. The relationships that matter: social network site use and social wellbeing among older adults in the United States of America. Ageing and Society. 2015;36(9):1826-52. doi: 10.1017/s0144686x15000677. (Page 16, Line 323)*

*41.	Wu H-Y, Chiou A-F. Social media usage, social support, intergenerational relationships, and depressive symptoms among older adults. Geriatric Nursing. 2020;41(5):615-21. doi:* [*https://doi.org/10.1016/j.gerinurse.2020.03.016*](https://doi.org/10.1016/j.gerinurse.2020.03.016)*. (Page 16, Line 323)*

*44.	Hu Y, Qian Y. COVID-19, Inter-household Contact and Mental Well-Being Among Older Adults in the US and the UK. Frontiers in Sociology. 2021;6. doi: 10.3389/fsoc.2021.714626. (Page 16, Line 331)*

*45.	Choi NG, DiNitto DM, Marti CN, Choi BY. Telehealth Use Among Older Adults During COVID-19: Associations With Sociodemographic and Health Characteristics, Technology Device Ownership, and Technology Learning. Journal of Applied Gerontology. 2022;41(3):600-9. doi: 10.1177/07334648211047347. PubMed PMID: 34608821. (Page 16, Line 341)*

*46.	Vroman KG, Arthanat S, Lysack C. “Who over 65 is online?” Older adults’ dispositions toward information communication technology. Computers in Human Behavior. 2015;43:156-66. doi:* [*https://doi.org/10.1016/j.chb.2014.10.018*](https://doi.org/10.1016/j.chb.2014.10.018)*. (Page 16, Line 342)*
