# Supplementary material for: Acceptance of communication technology, emotional support and subjective well-being for Chinese older adults living alone during COVID-19: A moderated mediation model
Source: PLoS One. 2023 Sep 21;18(9):e0291806. doi: 10.1371/journal.pone.0291806 (PMC10513265; doi:10.1371/journal.pone.0291806)
Supplement: Attachment — Submitted filename: The result of review PONE-D-22-27277.docx [file pone.0291806.s004.docx]

The result of review PONE-D- D-22-27277
Acceptance of Communication Technology, Emotional Support and Subjective Wellbeing for Chinese Older Adults Living Alone during COVID-19: A Moderated Mediation

Model.

This article is good, but there are some notes for improvement to make it better. First, the abstract must be corrected, where the abstract contains research methods. Second, explain in more detail along with the reasons why it is important to research your research study. Third, describe the research gap, between the current state and the desired condition, Fourth, explain what are the benefits for further research. Fifth, add journals from similar previous studies. Passion for improvement. Goodluck.
