# Supplementary material for: Acceptance of communication technology, emotional support and subjective well-being for Chinese older adults living alone during COVID-19: A moderated mediation model
Source: PLoS One. 2023 Sep 21;18(9):e0291806. doi: 10.1371/journal.pone.0291806 (PMC10513265; doi:10.1371/journal.pone.0291806)
Supplement: Attachment — Submitted filename: Response to Reviewers.docx [file pone.0291806.s005.docx]

**Response to Editor Comments:**

**1. Please review your reference list to ensure that it is complete and correct. If you have cited papers that have been retracted, please include the rationale for doing so in the manuscript text, or remove these references and replace them with relevant current references. Any changes to the reference list should be mentioned in the rebuttal letter that accompanies your revised manuscript. If you need to cite a retracted article, indicate the article’s retracted status in the References list and also include a citation and full reference for the retraction notice.**

***The full reference list has been checked and updated accordingly. We have included the following papers in response to the reviewer’s comments.***

16. Rolandi, E., Vaccaro, R., Abbondanza, S., Casanova, G., Pettinato, L., Colombo, M., & Guaita, A. (2020). Loneliness and social engagement in older adults based in Lombardy during the COVID-19 lockdown: The long-term effects of a course on social networking sites use. *International journal of environmental research and public health*, *17*(21), 7912. (Page 4, line 88)

17. Li, Y., Godai, K., Kido, M. *et al.* Cognitive decline and poor social relationship in older adults during COVID-19 pandemic: can information and communications technology (ICT) use helps?. *BMC Geriatr* **22**, 375 (2022). <https://doi.org/10.1186/s12877-022-03061-z> (Page 4, line 89)

18. Juvonen, J., Schacter, H. L., & Lessard, L. M. (2021). Connecting electronically with friends to cope with isolation during COVID-19 pandemic. *Journal of Social and Personal Relationships*, *38*(6), 1782-1799. (Page 4, line 92)

19. Foong, H. F., Lim, S. Y., Rokhani, F. Z., Bagat, M. F., Abdullah, S. F. Z., Hamid, T. A., & Ahmad, S. A. (2022). For better or for worse? A scoping review of the relationship between internet use and mental health in older adults during the COVID-19 pandemic. *International Journal of Environmental Research and Public Health*, *19*(6), 3658. (Page 4, line 94)

26. Dura-Perez E, Goodman-Casanova J, Vega-Nuñez A, Guerrero-Pertiñez G, Varela-Moreno E, Garolera M, Quintana M, Cuesta-Vargas A, Barnestein-Fonseca P, Gómez Sánchez-Lafuente C, Mayoral-Cleries F, Guzman-Parra J. The Impact of COVID-19 Confinement on Cognition and Mental Health and Technology Use Among Socially Vulnerable Older People: Retrospective Cohort Study. J Med Internet Res 2022;24(2):e30598 URL: <https://www.jmir.org/2022/2/e30598>. DOI: 10.2196/30598 (Page 5, line 117)

**2. Despite being good, this article could be improved a bit. The reviewer's comments must be corrected on some important parts of the manuscript, there are no gaps in the research, and the research methods are available.**

We thank the editor for the comments. We have addressed the comments below.

a) **Identification of the research gap**

***We have more clearly identified the research gap, by stating what is available in the current literature and what is missing, along with the information we desire to gain from conducting this research. We have inserted the following portion in the manuscript in page 6, line 135 to 143.***

*“This study aims to close two main research gaps. Other studies have examined the relationship between CT acceptance and competency on CT use [18], as well as CT use and emotional support and SWB. Much of this research focused on older adults without examining the mediating effect of living arrangements. Hence, this study aims to assess the association between CT acceptance (competency) and CT use specifically amongst older adults living alone. Additionally, there is little evidence in the current literature linking CT acceptance and competency with emotional support and SWB. Hence the study also aims to determine the association between CT acceptance (competency) and their roles in providing emotional support and SWB amongst older adults living alone.”*

b) **Research methods in the abstract section**

***We have edited the abstract, particularly the research methods section to ensure its alignment with the edited portions of the manuscript. The following insert has been added to the abstract on Page 2, line 37 and 42.***

*“Participants were surveyed from September to November 2020... PROCESS Model 1 was used to estimate the conditional effects of CT acceptance (competency) on emotional support for those living alone versus with others. Following which, PROCESS Model 7 was used to estimate the conditional indirect effects of CT acceptance (competency) on SWB through emotional support.”*

**Response to Reviewers’ Comments**

**Reviewer #2: This article is good, but there are some notes for improvement to make it better. First, the abstract must be corrected, where the abstract contains research methods. Second, explain in more detail along with the reasons why it is important to research your research study. Third, describe the research gap, between the current state and the desired condition, Fourth, explain what are the benefits for further research. Fifth, add journals from similar previous studies. Passion for improvement. Goodluck.**

Thank you for your comments. We hope we have responded to your comments to satisfactorily.

**1a) Revised abstract to include research methods**

***We have edited the abstract, particularly the research methods section to ensure its alignment with the edited portions of the manuscript. The following insert has been added to the abstract on Page 2, line 37 and 42.***

*“Participants were surveyed from September to November 2020... PROCESS Model 1 was used to estimate the conditional effects of CT acceptance (competency) on emotional support for those living alone versus with others. Following which, PROCESS Model 7 was used to estimate the conditional indirect effects of CT acceptance (competency) on SWB through emotional support.”*

**1b) Importance of conducting the current research**

***We have included additional reasons regarding the importance of our research study in the manuscript. The following inserts have been included on Page 6, line 143 and 147.***

*“Both pieces of information will be important to provide support to community care organisations and policymakers on how to address resistance and inertia amongst older adults living alone beyond the pandemic period and their daily lives. The information will also be useful in designing interventions to encourage older adults to use CT.”*

**1c) Describe the research gap, between the current state and desired condition.**

***We have more clearly identified the research gap, by stating what is available in the current literature and what is missing, along with the information we desire to gain from conducting this research. We have inserted the following portion in the manuscript in page 6, line 135 to 143.***

*“This study aims to close two main research gaps. Other studies have examined the relationship between CT acceptance and competency on CT use [18], as well as CT use and emotional support and SWB. Much of this research focused on older adults without examining the mediating effect of living arrangements. Hence, this study aims to assess the association between CT acceptance (competency) and CT use specifically amongst older adults living alone. Additionally, there is little evidence in the current literature linking CT acceptance and competency with emotional support and SWB. Hence the study also aims to determine the association between CT acceptance (competency) and their roles in providing emotional support and SWB amongst older adults living alone.”*

**1d) What are the benefits for further research.**

***The benefits for further research have been included in the manuscript, Page 19, line 379 to 401.***

*“Developing an understanding of the types of CT and their frequency of use could provide information to develop targeted interventions to encourage specific types of CT amongst older adults to improve their SWB… However, future research should aim to identify reasons for lack of social engagement to inform policies that can benefit this group of older adults. Accurate identification of these reasons could aid the respective support groups in understanding and targeting their respective needs and increase their social engagement and support from the community.”*

**1e) Addition of new literature**

***In response to the reviewer’s comments, we have included a list of literature from similar studies.***

16. Rolandi, E., Vaccaro, R., Abbondanza, S., Casanova, G., Pettinato, L., Colombo, M., & Guaita, A. (2020). Loneliness and social engagement in older adults based in Lombardy during the COVID-19 lockdown: The long-term effects of a course on social networking sites use. *International journal of environmental research and public health*, *17*(21), 7912. (Page 4, line 88)

*Rolandi et al., (2020) did a telephone survey on older adults aged 81 to 85, who previously participanted in a study aimed at evaluating the impact of social networking sites (SNSs) on self-perceived loneliness and social engagement with family and friends. Results suggest that participants who were trained to use SNS reported reduced feelings of being left out and lighter reduction in social contacts during COVID-19, as compared to participants who were not trained to use SNS.*

17. Li, Y., Godai, K., Kido, M. *et al.* Cognitive decline and poor social relationship in older adults during COVID-19 pandemic: can information and communications technology (ICT) use helps?. *BMC Geriatr* **22**, 375 (2022). <https://doi.org/10.1186/s12877-022-03061-z> (Page 4, line 89)

*Li et al., (2022) found that amongst older adults who were 80 years and above, non-information and communication technology (ICT) use was associated with cognitive decline, moderated by social isolation. They believe that for older adults who were vulnerable to poor social relationships, ICT use is potentially an efficient medium.*

18. Juvonen, J., Schacter, H. L., & Lessard, L. M. (2021). Connecting electronically with friends to cope with isolation during COVID-19 pandemic. *Journal of Social and Personal Relationships*, *38*(6), 1782-1799. (Page 4, line 92)

*Juvonen et al., (2021) conducted a cross-sectional survey with a sample of 295 participants (aged 18 to 70). Participants of all ages reported greater satisfaction with video calls during the imposed isolation. Overall findings highlight the potential psychological benefits of connecting electronically with close others, and suggest that connecting with friends offers a way to cope with imposed isolation as long as individuals are satisfied with their exchanges.*

19. Foong, H. F., Lim, S. Y., Rokhani, F. Z., Bagat, M. F., Abdullah, S. F. Z., Hamid, T. A., & Ahmad, S. A. (2022). For better or for worse? A scoping review of the relationship between internet use and mental health in older adults during the COVID-19 pandemic. *International Journal of Environmental Research and Public Health*, *19*(6), 3658. (Page 4, line 94)

*Foong et al., (2022) is a scoping review of the relationship between internet use and mental health in older adults during the COVID-19 pandemic. Their results suggest that Internet use for communication purposes seems to be associated with better mental health in older adults during COVID-19 pandemic.*

26. Dura-Perez E, Goodman-Casanova J, Vega-Nuñez A, Guerrero-Pertiñez G, Varela-Moreno E, Garolera M, Quintana M, Cuesta-Vargas A, Barnestein-Fonseca P, Gómez Sánchez-Lafuente C, Mayoral-Cleries F, Guzman-Parra J. The Impact of COVID-19 Confinement on Cognition and Mental Health and Technology Use Among Socially Vulnerable Older People: Retrospective Cohort Study. J Med Internet Res 2022;24(2):e30598 URL: <https://www.jmir.org/2022/2/e30598>. DOI: 10.2196/30598 (Page 5, line 117)

*Dura-perez et al., (2022) explored the impact of the COVID-19 outbreak on older adults’ mental well-being (e.g. quality of life, depression). The study also analyzed the association with technophilia (positive and enthusiasm towards technology) on the outcomes. Results suggest that older adults with higher technophilia were associated with better mental outcomes.*

***In addition, we would like to highlight that we have already cited literature that examined similar areas of research in the current version of the manuscript. In the introduction, the following papers with a short description of each paper’s content, are included:***

4. Fingerman KL, Ng YT, Zhang S, Britt K, Colera G, Birditt KS, et al. Living alone during COVID-19: Social contact and emotional well-being among older adults. J Gerontol B Psychol Sci Soc Sci. 2021;76(3):e116–e21. doi: doi:10.1093/geronb/gbaa200. (Page 3, Line 62)

*Fingerman et al., (2021) examined how living alone was associated with daily social contact and emotional well being among older adults during the pandemic. Results suggest that older adults who live alone were less likely to see others to receive or provide help. In-person contact was associated with more positive emotions and less negative emotions for older adutls living alone, as compared to phone contact.*

6. Noguchi T, Hayashi T, Kubo Y, Tomiyama N, Ochi A, Hayashi H. Living Alone and Depressive Symptoms among Older Adults in the COVID-19 Pandemic: Role of Non–Face-to-Face Social Interactions. Journal of the American Medical Directors Association. 2023;24(1):17-21.e4. doi: <https://doi.org/10.1016/j.jamda.2022.10.014>. (Page 4, line 90)

*Noguchi et al., (2023) examined the association of living alone with changes in depressive symptom status, and the moderating effect of non-face-to-face social interactions (e.g. phone calls, emails) among older adults during the COVID-19 pandemic. Results suggest a protective effect of weekly non-face-to-face social interactions for depressive symptom onset for older adults living alone.*

11. Chopik WJ. The benefits of social technology use among older adults are mediated by reduced loneliness. Cyberpsychology, Behavior and Social Networking. 2016;19(9). doi: 10.1089/cyber.2016.0151. (Page 3, line 76)

*Chopik (2016) examined the assoiation between technology use for social reasons and psychological health amongst older adults. Results suggest that high levels of social technology use was associated with higher subjective well-being and fewer depressive symptoms. Results also suggest that technology has the potential to cultivate successful relationships among older adults.*

12. Sims T, Reed AE, Carr DC. Information and communication technology use is related to higher well-being among the oldest-old. J Gerontol B Psychol Sci Soc Sci. 2017;72(5):761–70. doi: doi:10.1093/geronb/gbw130. (Page 3, line 76)

*Sims et al., (2017) examined the use of information and communiation technology (ICT) in relation to social goals and higher well-being amongst the oldest-old. Results suggest that ICT use predicted higher well-beig across outcomes, mediated by social motivations to connect with others).*

13. Holtzman S, DeClerck D, Turcotte K, Lisi D, Woodworth M. Emotional support during times of stress: Can text messaging compete with in-person interactions? Computers in Human Behavior. 2017;71:130-9. doi: <https://doi.org/10.1016/j.chb.2017.01.043>. (Page 3, line 78)

*Holtzman et al., (2017) compared the impact of in-person support to text messaging support. Results sgugest that in-erson support resulted in higher levels of positive emotions than texting. However, when interacting with close friends, satisfaction for both modes of communication was equivalent.*

14. Li J, Zhou X. Internet use and Chinese older adults’ subjective well-being (SWB): The role of parent-child contact and relationship. Computers in Human Behavior. 2021;119:106725. doi: <https://doi.org/10.1016/j.chb.2021.106725>. (Page 4, line 83)

*Li et al., (2021) examines whether the frequency of parent-child contact and parent-child relationship can medicate the relationship between Internet use and Chinese older people’s subjective well-being. Results suggest that Internet use was associated with older adults’ subjective well-being, mediated by parent-child contact and relationship. Findings further suggest that Internet use may empower older adults to maintain close intergenerational relationships contributing to their subjective well-being.*

15. Kotwal AA, Holt-Lunstad J, Newmark RL, Cenzer I, Smith AK, Covinsky KE, et al. Social Isolation and Loneliness Among San Francisco Bay Area Older Adults During the COVID-19 Shelter-in-Place Orders. J Am Geriatr Soc. 2021;69(1):20-9. Epub 20201009. doi: 10.1111/jgs.16865. PubMed PMID: 32965024; PubMed Central PMCID: PMCPMC7536935. (Page 4, line 86)

*Kotwal et al., (2021) aimed to investigates experiences of social isolation and loneliness during shelter-in-place orders, as well as unmet health needs related to changes in social interactions. Results suggest that most participants communicated via telephone interactions. Results from open-ended feedback by participant further suggest that access to technology was central to their ability to cope with restrictions and maintain social connections.*

***In the discussion section, the following papers are included:***

45. Yu RP, McCammon RJ, Ellison NB, Langa KM. The relationships that matter: social network site use and social wellbeing among older adults in the United States of America. Ageing and Society. 2015;36(9):1826-52. doi: 10.1017/s0144686x15000677. (Page 17, line 340)

*Yu et al., (2015) studied how the use of social network sites (SNSs) was associated with social wellbeing outcomes. Results suggest that SNS use is positively associated with non-kin related social wellbeing outcomes, including perceived support and connectedness from friends.*

46. Wu H-Y, Chiou A-F. Social media usage, social support, intergenerational relationships, and depressive symptoms among older adults. Geriatric Nursing. 2020;41(5):615-21. doi: <https://doi.org/10.1016/j.gerinurse.2020.03.016>. (Page 16, line 340)

*Wu & Chiou (2020) examined using a cross-sectional, correlational study design on social media usage, social support, intergenerational relationships and depressive symptoms. Their findings suggest that social media usage and social support were negatively associated with depressive symptoms. They recommend promoting the use of social media to increase social interactions amongst older adults.*

49. Hu Y, Qian Y. COVID-19, Inter-household Contact and Mental Well-Being Among Older Adults in the US and the UK. Frontiers in Sociology. 2021;6. doi: 10.3389/fsoc.2021.714626. (Page 17, line 348)

*Hu & Qian (2021) examined how inter-household contact in face-to-face and virtual forms (separately and in combination) related to older adults’ mental well-being during the pandemic. Study findings suggest the importance of social communication (especially face-to-face contact) in sustaining older adults’ mental well-being.*

***If the reviewer has any suggestions for relevant study, please feel free to recommend them to use for consideration.***
